# Prevalence and factors associated with Hepatitis B infection in the general population: a cross-sectional study in Moshi municipality, North-Eastern Tanzania

**DOI:** 10.4314/ahs.v25i2.5

**Published:** 2025-06

**Authors:** Elichilia R Shao, Innocent B Mboya, Jeremia J Pyuza, Florida Muro, Daniel Gunda, Harold L Mashauri, Semvua Kilonzo, Christian Issangya, Deborah Mchaile, Benjamin Shayo, Ephata Kaaya, Venance P Maro, Sarah Urasa, Gileard Masenga

**Affiliations:** 1 Department of Internal Medicine, KCMC University, P.O. Box 2240, Moshi, Tanzania; 2 Department of Internal Medicine, Kilimanjaro Christian Medical Centre, P.O. Box 3010, Moshi, Tanzania; 3 School of Public Health, KCMC University, P.O. Box 2240, Moshi Tanzania; 4 Mega-Afya and Business Company Limited, P.O. Box 6791, Moshi, Tanzania; 5 Better Human Health Foundation, P.O. Box 1348, Moshi, Tanzania; 6 Africa Academy for Public Health, P.O. Box 32273, Dar es Salaam, Tanzania; 7 Department of Pathology, KCMC University, P.O. Box 2240, Moshi Tanzania; 8 Catholic University of Health and Allied Science, P.O. Box 1464, Mwanza, Tanzania; 9 Department of General Surgery, KCMC University, P.O. Box 2240, Moshi, Tanzania; 10 Department of Paediatric and Child Health, KCMC University, P.O. Box 2240, Moshi, Tanzania; 11 Department of Obstetrics and Gynaecology, KCMC University, P.O. Box 2240, Moshi, Tanzania

**Keywords:** Hepatitis B, general population, Tanzania

## Abstract

**Background:**

Hepatitis B virus (HBV) infection is a significant public health problem, even with the presence of affordable and effective vaccination. Toward hepatitis B elimination by 2030, it is essential to know the prevalence of HBV in the general population and associated risk factors so as to set priorities for specific prevention and control strategies in this population.

**Methods:**

This was an analytical cross-sectional study among 1399 general population aged 18 years and above who came for HBV screening during the world viral hepatitis day on 28th and 29th July 2019. Serological marker of HBV was done using Determine HBsAg 2 Rapid Test. Data were cleaned and analyzed using STATA version 15.1. Log-binomial regression was used to estimate odds ratios (ORs) and 95% confidence intervals (CIs) for factors associated with HBV infection.

**Results:**

The mean age of all study participants was 43.3±16.1 years, 57.4% were aged 40 years and above, and 57.8% were females. The positive rate of hepatitis B surface antigen (HBsAg) was 2.4%. Significantly higher odds of HBV infection were among respondents aged 30-39 (OR=2.70, 95%CI 1.22, 5.97, p=0.01) compared to those aged 40 years and above and those with a history of HBV infection (OR=8.46, 95%CI 2.66, 26.86, p<0.001).

**Conclusion:**

The prevalence of hepatitis B infection is at an intermediate epidemic level in a semi-urban population in North-eastern Tanzania. Particular attention should be among middle-aged adults, such as those aged 30-39 years compared to their older counterparts and those with a history of HBV infection.

## Introduction

Hepatitis B virus (HBV) infection is a significant public health problem, even with the presence of affordable and effective vaccination. Worldwide, in 2019, 296 million people were estimated to live with chronic HBV, with 1.5 million new infections each year^1^. Among those infected, the majority do not know their status^2^,^3^. HBV elimination strategies by 2030 need collaborative efforts by all stakeholders to respond towards sustainable development goal 3.3 on combating hepatitis. The main target is to reduce new HBV infections by 90% and mortality by 65%^2^,^3^. Nearly half of the global population lives in high prevalence areas. Globally, the prevalence of HBV infection is 1.3%, and ranges from 0.2% in America to 3% in Africa^4^. However, Asia and sub-Saharan Africa carries the highest burden, where more than 5% of the adults in the general population are chronically infected with HBV^5^. In East Africa, the prevalence of HBsAg was approximated to be 8%^6^. The prevalence of Hepatitis B infection in Tanzania differs across different groups with the overall average estimated to be about 7%, which is also similar to that among health care workers in the country^7^. Studies in specific populations, such as blood donors in Dar es Salaam, the capital city of Tanzania, reported a slightly higher prevalence (8.8%)^8^. These estimates put Tanzania among the high endemic countries; hence extra-efforts toward elimination are necessary.

Tanzania established pediatric vaccination campaigns in the year 2002^9^,^10^ to address different infectious diseases including HBV infection. Viral hepatitis elimination strategies should go hand in hand with enough data from the diversity of the population to build up a case on preventive measures. During World Hepatitis Day, screening services for HBV were made available among adults aged 18 years and above in Moshi Municipality, Northern Tanzania, for two days.

Several factors have been found to be associated with Hepatitis B infection in the general population. One study in Uganda reported that^11^–^16^. HBV infection has been associated with tattooing, family history of HBV infection, history of hospitalization or surgery and history of blood transfusion^1^,^16^–^19^. In Tanzania, it was reported that HBV infection was the most frequent Transfusion Transmitted infection^8^,^13^. Vaccination against HBV infection has been shown to be protective significantly against HBV infection^1^,^15^.

Toward hepatitis B elimination by 2030, it is essential to know the prevalence of HBV in the general population and associated risk factors, especially in high endemic areas. This study aimed to determine the prevalence of HBV infection and associated factors in the general population in Moshi Municipality, Northern Tanzania during the world hepatitis day in 28th July, 2019. Findings from this study will help the country to prioritize specific prevention and control strategies in this population.

## Methods

This cross-sectional survey among adults aged 18 years and above in Moshi Municipality, North-eastern Tanzania, was conducted during the World Hepatitis Day on 28th and 29th July 2019, at Kilimanjaro Christian Medical University College (KCMUCo). The sample size consisted of 1399 consenting adults aged 18 years or older who came out to be screened for hepatitis B virus infection participated in this study. All individuals who turned up for screening during the two consecutive days were enrolled in the study.

The dependent variable in this study was HBV infection based on the serological results. We considered the positive rate of HBsAg to be the prevalence of hepatitis B. Serological marker of HBV was done using Determine HBsAg 2 rapid test, which has high specificity (99.6%) and sensitivity (97.2%) (20) . Independent variables included background characteristics and the exposure to the risks of HBV infection. The background characteristics included age categories (years) (≤29, 30-39, 40+), sex (male, female), marital status (single, married/Cohabiting, widow/divorced), area of residence (rural, urban, education level (primary, secondary, college/university), and occupation (none, farmer/Business, employed). Exposure to the risk of HBV infection included history of blood transfusion, surgery, blood splash to the eyes or mouth, intravenous injection, intramuscular injection, an invasive procedure such as endoscopy, all coded as Yes/No. Furthermore, the risks included sharing a blade for nail cutting, working in a hospital/nursing home/child center, having a relative ever diagnosed with hepatitis B in the household, having sex with multiple partners, history of Hepatitis B diagnosis and ever received Hepatitis B vaccination, also coded as Yes/No. Knowledge of HBV infection was assessed using 15-item questions that required Yes/No responses. The total scores were multiplied by 100 and those who scored <50% were considered to have poor knowledge, and good if otherwise. Trained medical doctors collected the data using an electronic questionnaire that included closed and open-end questions. Interviews were conducted in a private room following informed consent from all study participants. Immediately after the HBV testing using a rapid test, participants received their test results and counseling, including follow-up assessments in nearby health facilities.

All collected data were double entered into the excel sheet by two independent staff, which were later managed, cleaned and analyzed using Stata version 15.1. Numeric variables were summarized using mean and standard deviation and categorical variables using frequencies and percentages. We used the Chi-squared (χ2) test to compare the proportion of HBV infection by respondent characteristics and the pairwise correlation to assess collinearity between variables entered in the regression models. The log-binomial regression model (i.e., a generalized linear model with binomial family and log-link function) was used to estimate the odds ratio (OR) and 95%CI for factors associated with HBV infection. Stepwise regression was used for variable selection and model building. Using a 10% significant level, participant age, sex, history of HBV infection, and vaccine uptake were retained in the model. We further explored the effect of keeping sex in the model in which the model without this variable was considered the best fit. To test nested models, we used the likelihood ratio test. After controlling for potential confounders, we considered variables with p<0.05 to be statistically associated with the odds of HBV infection.

There was no patient and public involvement in this study. All participants who participated in this study were given their blood results at the end of the interview accompanied with counseling on follow-up at the respective hospitals for further assessment and treatment if eligible.

Ethical clearance was sought and granted by the Kilimanjaro Christian Medical College Research and Ethics Review Committee (KCMU-CRERC) with reference number 916. Written consent was obtained from every participant who was involved in this study. Interviews were conducted in private rooms to ensure privacy during interviews, while unique identification numbers were used to ensure anonymity and confidentiality of participant information. All questionnaires and participant data were stored in a secure room at KCMC hospital and in a password-protected computer, respectively.

## Results

### Participant background characteristics

A total of 1399 adults aged 18 years or older from the general population in Moshi Municipality took part in this study (Table 1). Their mean age ± standard deviation was 43.3±16.1, and 57.4% were aged 40 years or older. Nearly two-thirds of all participants were females, 57.8% were married or cohabiting with their partners, 64% resided in urban areas, 41.3% had primary education level, and 45.5% engaged in farming or business activities.

**Table 1:** Respondent characteristics (N=1399)

Variable	Frequency	Percentage
Age categories (years) *		
Mean ± SD	43.3±16.1	
≤29	348	24.9
30-39	247	17.7
40+	803	57.4
Sex*		
Male	493	35.3
Female	905	64.7
Marital status*		
Single	491	35.3
Married/Cohabiting	804	57.8
Widow/Divorced	97	7
Area of residence*		
Rural	501	36.1
Urban	887	63.9
Education level*		
Primary	568	41.3
Secondary	392	28.5
College/University	414	30.1
Occupation*		
None	260	18.9
Farmer/Business	628	45.5
Employed	491	35.6

* Frequencies do not tally to the total due to missing values in these variables.

### Exposure to the risk of HBV infection

Among all participants in this study, 8.7% had a history of blood transfusion, 30% had a history of surgery, 6.8% had a history of blood splashes to the eyes or mouth, and 71.2% had a history of intravenous injections. Furthermore, more than half (54%) had a history of intramuscular injections, 22.6% ever shared a blade for nail cutting, 8% had a history of invasive procedure such as endoscopy, and 4.5% had a relative with a history of HBV diagnosis. Furthermore, about a quarter (24.4%) of all respondents have had sex with multiple sexual partners, 1.6% had been diagnosed with HBV infection in the past and had received HBV, respectively. Sixty percent of all respondents had good knowledge about HBV infection. The prevalence of HBV infection was only 2.4% (Table 2).

**Table 2:** Exposure to the risk of HBV infection (N=1399)

Variable	Frequency	Percentage
History of blood transfusion*		
Yes	122	8.7
No	1275	91.3
History of surgery*		
Yes	423	30.3
No	975	69.7
History of blood splash to the eyes or mouth*		
Yes	95	6.8
No	1302	93.2
History of intravenous injection*		
Yes	996	71.2
No	402	28.8
History of intramuscular injection*		
Yes	752	53.9
No	642	46.1
**Ever shared a blade for nail cutting**		
Yes	316	22.6
No	1083	77.4
History of any invasive procedure such as endoscopy*		
Yes	112	8.0
No	1284	92
Work in a hospital/nursing home/child center*		
Yes	266	19.1
No	1130	80.9
**A relative ever diagnosed with hepatitis B in the household**		
Yes	63	4.5
No	1334	95.5
Ever had sex with multiple partners*		
Yes	341	24.4
No	1056	75.6
Ever been diagnosed with Hepatitis B*		
Yes	23	1.6
No	1374	98.4
**Ever received Hepatitis B vaccination**		
Yes	22	1.6
No	1377	98.4
**HBV knowledge**		
Poor	570	40.7
Good	829	59.3
**HBV status**		
Positive	34	2.4
Negative	1365	97.6

* Frequencies do not tally to the total due to missing values in these variables.

### Unadjusted analysis for factors associated with HBV infection

Results from the crude/unadjusted showed that respondent age (years) and history of HBV infection were the only factors associated with positive serology status (Table 3 and 4). The prevalence of HBV infection was about 5% among those aged 30-39 years and 2.3% among those aged <29 years. Significantly higher odds of HBV infection were among respondents aged 30-39 years (OR=2.79, 95%CI 1.31, 5.95, p=0.01) compared to those aged 40 years and above. No significant association observed between HBV infection and other demographic characteristics in this study (Table 3).

**Table 3:** Unadjusted analysis for the association between HBV infection and demographic characteristics

Variables	Total	+Ve Serology n (%)	COR† (95%CI)	p-value
Age categories (years) *				
≤29	348	8 (2.3)	1.32 (0.56, 3.11)	0.53
30-39	247	12 (4.9)	2.79 (1.31, 5.95)	0.01
40+	803	14 (1.7)	1.00	
Sex*				
Male	493	15 (3.0)	1.00	
Female	905	19 (2.1)	0.69 (0.35, 1.35)	0.28
Marital status*				
Single	491	15 (3.1)	1.00	
Married/Cohabiting	804	17 (2.1)	0.69 (0.35, 1.37)	0.29
Widow/Divorced	97	2 (2.1)	0.67 (0.16, 2.90)	0.60
Area of residence*				
Rural	501	14 (2.8)	1.24 (0.63, 2.43)	0.53
Urban	887	20 (2.3)	1.00	
Education level*				
Primary	568	11 (1.9)	1.00	
Secondary	392	13 (3.3)	0.80 (0.34, 1.87)	0.61
College/University	414	10 (2.4)	1.37 (0.61, 3.09)	0.45
Occupation*				
None	260	4 (1.5)	1.00	
Farmer/Business	628	16 (2.5)	1.66 (0.56, 4.91)	0.36
Employed	491	14 (2.9)	1.85 (0.62, 5.57)	0.27

* Frequencies do not tally to the total due to missing values in these variables.

† COR: Crude odds ratio.

**Table 4:** Unadjusted analysis for the association HBV infection and exposure to potential risk factors

Variable	Total	+Ve Serology n (%)	COR* (95%CI)	p-value
History of blood transfusion*				
Yes	122	1 (0.8)	0.32 (0.04, 230)	0.25
No	1275	33 (2.6)	1.00	
History of surgery*				
Yes	423	7 (1.7)	0.60 (0.26, 136)	0.22
No	975	27 (2.8)	1.00	
History of blood splash to the eyes or mouth*				
Yes	95	2 (2.1)	0.86 (0.21, 352)	0.83
No	1302	32 (2.5)	1.00	
History of intravenous injection*				
Yes	996	23 (2.3)	0.84 (0.42, 1.71)	0.64
No	402	11 (2.7)	1.00	
History of intramuscular injection*				
Yes	752	16 (2.1)	0.76 (0.39, 1.48)	0.42
No	642	18 (2.8)	1.00	
**Ever shared a blade for nail cutting**				
Yes	316	11 (3.5)	1.64 (0.81, 333)	0.17
No	1083	23 (2.1)	1.00	
History of any invasive procedure such as endoscopy*				
Yes	112	2 (1.8)	0.72 (0.107, 295)	0.64
No	1284	32 (2.5)	1.00	
Work in a hospital/nursing home/children center*				
Yes	266	7 (2.6)	1.10 (0.48, 2.50)	0.82
No	1130	27 (2.4)	1.00	
**Have a relative diagnosed with hepatitis B in the household**				
Yes	63	3 (4.8)	2.05 (0.64, 652)	0.23
No	1334	31 (2.3)	1.00	
Ever had sex with multiple partners*				
Yes	341	10 (2.9)	1.29 (0.62, 2.67)	0.49
No	1056	24 (2.3)	1.00	
Ever been diagnosed with Hepatitis B*				
Yes	23	4 (17.4)	7.97 (3.05,. 20.77)	<0.001
No	1374	30 (2.2)	1.00	
Ever received is Hepatitis B vaccination*				
Yes	22	2 (9.1)	3.91 (1.00, 15.32)	0.05
No	1377	32 (2.3)	1.00	
**HBV knowledge**				
Good	829	20 (2.4)	0.98 (0.50, 1.93)	0.96
Poor	570	14 (2.5)	1.00	

* Frequencies do not tally to the total due to missing values in these variables.

† COR: Crude odds ratio.

The association between HBV infection and exposure to potential risk factors is in Table 4. A significantly higher prevalence of HBV infection was observed among respondents with a history of HBV infection (17.4%) compared to 2.2% who did not. Only two (9.1%) of 22 respondents who had received HBV vaccination had positive serology status. Participants with a self-reported history of HBV infection had about eight-fold higher odds of having seropositive status (OR=7.97, 95%CI 3.05, 20.77, p<0.001) compared to those who did not. Good knowledge of HBV infection reduced the odds of infection (OR=0.98, 95%CI 0.50, 1.93), but this association was not statistically significant (p=0.96).

### Adjusted analysis for factors associated with HBV infection

After adjusting for other variables at a 5% significance level, the factors associated with seropositivity were age categories (years) and a history of HBV infection. Respondents aged 30-39 years had 2.7 times higher odds of HBV infection (OR=2.70, 95%CI 1.22, 5.97, p=0.01) compared to those aged 40 years and above. Those with a history of HBV infection had over eight times higher odds of seropositivity (OR=8.46, 95%CI 2.66, 26.86, p<0.001) (Table 5).

**Table 5:** Adjusted analysis for factors associated with HBV infection

Variables	AOR*	95% CI	p-value
**Age categories (years)**			
≤29	1.32	(0.55,3.19)	0.54
30-39	2.70	(1.22,5.97)	0.01
40+	1.00		
**Ever been diagnosed with Hepatitis B**			
Yes	8.46	(2.66, 26.86)	<0.001
No	1.00		

* AOR: Adjusted odds ratio. Adjusted for age categories (years) and history of HBV infection.

## Discussion

The prevalence of hepatitis B infection in the general adult population in Moshi Municipality was 2.4% and was high among respondents aged 30-39 compared to those aged ≥40 years and those with a history of HBV infection. Prevalence in this study is slightly higher compared to the global prevalence of 1.3%^4^, but comparatively small to the prevalence in Africa (3%)^4^ and that in East Africa (8%)^6^. Globally, still this prevalence indicates the need for more efforts to address HBV infection in Tanzania among the general population.

Moreover, this prevalence is lower than the overall prevalence in Tanzania (6.9%) and that among community volunteers (8.8%)^7^. These differences might be explained by the possibility that individuals with known HBV infection status did not turn-up for screening during the World Hepatitis Day when the study was conducted in 2019. The HBV infection prevalence in this study also is more than twice lower compared to that among healthcare workers in Tanzania of 5.7%^21^ to 7%^22^, essentially because healthcare workers are the more risker group compared to the general population. Healthcare workers working environments increases chances of getting HBV infection almost four times to the general population^23^.

Respondents' age and history of HBV infection were the only factors associated with a higher prevalence of HBV seropositivity in this study. Individuals aged 30-39 years had higher chances of acquiring HBV infection than their counterparts. This finding is similar to the study done in Tanzania among healthcare workers^22^, among the general population in Uganda^11^,^12^, Ethiopia^16^, Iran^14^ and China^15^. Adult population might be more prone to HBV infection because of increased chances risk of multiple exposures such as medical procedures like surgery and blood transfusion, low social-economic status, and multiple sexual partners^13^–^18^. Moreover, respondents' history of previous HBV infection has been associated with the HBV infection in this study. This might be because the previous infection was not treated, and participants might have developed chronic HBV infection^1^.

Contrary to other studies^1^,^11^–^19^, there were no statistically significant association between HBV infection positive serology and participants' with other demographic characteristics and exposure to potential HBV risk factors in this study. It is possible that most of those who were familiar with their HBV infection status dinot participate during the screening day.

## Strength and limitation of the study

This was the first study to determine the prevalence of hepatitis B Virus infection among general population in the northern Tanzania, which constituted individuals with diverse characteristics and risk factor profiles, which might represent the general population in the region.

This study had several limitations. Firstly, we were not able to measure hepatitis B core antibody (HBcAb) to know how many participants were exposed. Secondly, for adults who turned positive, we could not measure their viral loads to determine their eligibility for treatment. Thirdly, the antibody titres were not taken to know the level of protection among the infected population. However, we primarily aimed to determine the prevalence of hepatitis B infection in our general population during the World Hepatitis Day. The findings from this study might not be generalized to other settings and other groups with high exposure to HBV risk factors such as healthcare workers, female sex workers, and people who inject drugs. Lastly, a very small number of +ve serology might have influenced the unobserved associations between HBV infection and potential risk factors in this study.

## Conclusion and Recommendation

The prevalence of hepatitis B infection is at an intermediate epidemic level in a semi-urban population in North-eastern Tanzania. The study recommends increasing HBV vaccination coverage among all eligible individuals, especially those at high-risk of infection. Early diagnosis and treatment coverage should be the primary focus toward elimination of HBV infection by 2030. Particular attention should be among middle-aged adults, specifically those aged 30-39 years compared to their older counterparts and the groups at high risk of infection in the general population. Furthermore, we recommend a larger nationwide study to provide updates on the prevalence, risk factors, and prevention strategies in the general population.
